# Prevalence of Central Line-Associated Bloodstream Infections (CLABSI) in Intensive Care and Medical-Surgical Units

**DOI:** 10.7759/cureus.22809

**Published:** 2022-03-03

**Authors:** Harjyot Toor, Saman Farr, Paras Savla, Samir Kashyap, Sharon Wang, Dan E Miulli

**Affiliations:** 1 Neurosurgery, Riverside University Health System Medical Center, Moreno Valley, USA; 2 Infectious Disease, Arrowhead Regional Medical Center, Colton, USA

**Keywords:** clabsi, central line, critical care, infectious disease, trauma

## Abstract

Background: Central line-associated bloodstream infections (CLABSIs) remain an important preventable healthcare-associated infection with a 2020 rate of 0.87 (per 1,000 central line days) in the United States intensive care units (ICU). Methods: This was a retrospective cohort study of all adult patients in our institution. The total number of central venous catheter (CVC) insertions and line days were determined using daily unit logs maintained by unit managers. Central line insertion practice (CLIP) compliance was calculated as the total number of CLIP forms submitted divided by the total number of newly-inserted CVCs with and without associated CLIP forms as determined by unit logs.

Results: A total of 1,125 CVCs were reviewed (448 - ICU and 677 - medical-surgical units). Of the 13 CLABSI, one patient had internal jugular (IJ), one patient had subclavian (SC), four patients had femoral, three patients had peripherally inserted central catheter (PICC) and four patients had hemodialysis catheters. Patients with CLABSI had CVC inserted for a range of five to 92 days with the average number of line days being 29 days.

Conclusion: Based on the analysis of our CLABSI patient population, we recommend our institution implement the following criteria to decrease the prevalence of CLABSI: All patients receiving a CVC must adhere to CLIP documentation in all units, any femoral or HD CVC placed without a CLIP form should have the line changed within 48 hours, those patients with a femoral CVC or hemodialysis catheter in place for four days or greater with an abnormal WBC (<4.0 or >11 mg/dL) or abnormal temperature (<97.0F or >100.4F) should be considered for catheter exchange, and those patients with an IJ, SC, or PICC CVC in place for seven days or greater with an abnormal WBC or abnormal temperature should have the catheter changed.

## Introduction

Central line-associated bloodstream infections (CLABSIs) remain an important, preventable healthcare-associated infection with a 2020 rate of 0.87 (per 1,000 central line days) in the United States intensive care units (ICU) [1-3]. CLABSIs carry a mortality rate of 12%-15% and an odds ratio of in-hospital death is as high as 2.75 [4]. CLABSIs are also associated with increased length of hospital stay and increased healthcare cost with each case accounting for approximately $46,000. Most cases of CLABSIs are preventable with proper aseptic techniques, surveillance, and management strategies.

The CDC’s “Guidelines for the Prevention of Intravascular Catheter-Related Infections” advocate evidence-based central line insertion practices (CLIPs) to reduce the risk of subsequent CLABSI. The sterile technique includes hand hygiene, maximal sterile barriers during insertion, use of an antiseptic prior to insertion with time to allow the antiseptic to dry before catheter insertion (CDC NHSN). Centers across the United States have implemented CLIP and CLIP adherence is under stringent institutional and federal supervision. Our goals were to: (1) assess the prevalence of CLABSI in both ICU and medical-surgical units (also known as the “floor”) at our institution and (2) assess whether CLIP sheet compliance decreases the incidence of CLABSI.

## Materials and methods

This was a retrospective cohort study of all adult patients in our institution, a Level 2 trauma center in Southern California, with an indwelling central venous catheter (CVC) of two or more days between January 1, 2020 and December 31, 2020. All patients were screened with ICD-10 Code T80.211A (CLABI) and those submitted by our institution to the National Healthcare Safety Network (NHSN).

CLABSI was defined as a laboratory-confirmed new bloodstream infection that is not secondary to an infection at another body site - in accordance with the CDC definition. Patient data were collected from unit log review and from the log submitted by our institution to the National Healthcare Safety Network (NHSN). Patients under 18 years, having a CVC placed prior to admission, negative blood cultures, open wounds/abrasions within 10 cm of CVC insertion, and evidence of sepsis and/or infection on admission were excluded. CVC documentation was reviewed in physical and electronic charts. Visual inspection and charted data on insertion date, body site location, and line type were compared to nursing electronic and paper CVC assessments. Patients missing any or all these metrics were excluded from the study.

Multiple attempts were made to collect from the electronic medical record the total number of CVC insertions performed at our institution using CPT codes and ICD-10 procedure codes but did not yield results. The total number of CVC insertions and line days were eventually determined using daily unit logs maintained by unit managers. CLIP compliance was calculated as the total number of CLIP forms submitted divided by the total number of newly inserted CVCs with and without associated CLIP forms as determined by unit logs. For the latter, missing forms were considered non-compliant. Comparisons were made using chi-squared analysis. Data were stratified by ICU/non-ICU location and CVC-type (subclavian [SC], internal jugular [IJ], femoral, peripherally inserted central catheter [PICC], or hemodialysis [HD]).

## Results

CLABSI prevalence**

**Figure 1:**
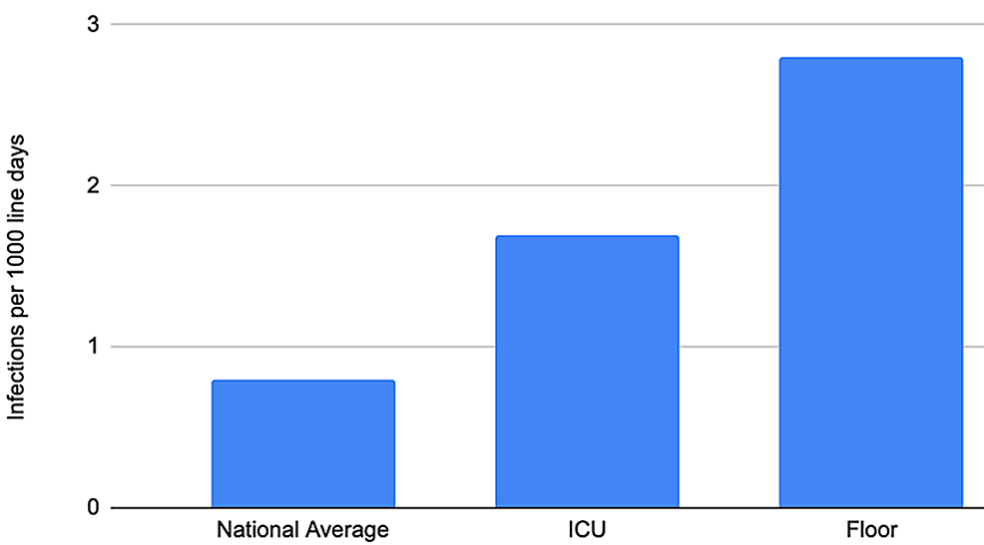
Institutional comparison of CLABSI rate to the national average Floor: Medical-Surgical Unit, CLABSI - Central line-associated bloodstream infection

A total of 1,125 CVC were reviewed (448 from ICU and 677 from medical-surgical units). Of those 1,125 central lines; 220 were IJ, 107 were SC, 188 were femoral, 449 were PICC, and 161 were HD catheters (Table 1). Fifty patients were diagnosed and treated for CLABSI at the time of discharge. Some patients were included more than once as they had multiple, separate hospitalizations. Thirty-two patients were excluded due to pre-hospital CVC insertion, one patient was excluded due to no growth on blood culture and four patients were excluded due to positive blood culture prior to two days (Tables 2, 3). The total number of days of exposure to CVCs (line days) among all patients with CLABSI was 379 days, ranging from five to 93 days. Of the 13 CLABSI, one patient had IJ, one patient had SC, four patients had femoral, three patients had PICC, and four patients had hemodialysis catheters (Table 4). CLABSI rate for the ICU was 1.7 (per 1,000 central line days) and for the medical-surgical units (or floor units) was 2.8 (per 1,000 central line days) (Figure 1). The highest incidences of CLABSI by type of CVC are femoral and hemodialysis (Table 4). When looking at the average line days to infection, it was 23.25 days for a CLABSI in the ICU and 31.6 days in the medical-surgical unit. When looking at the average line days without infection it was 5.3 days for a CVC without CLABSI in the ICU and 5.34 days in the medical-surgical unit (Table 5). 

**Table 1:** Type of central venous catheter insertion in ICU and medical-surgical patients PICC - peripherally inserted central catheter

Central Lines Placed 2020	Internal Jugular	Subclavian	Femoral	PICC	Hemodialysis	Total	Line Days
ICU	173	35	169	70	1	448	2,373
Medical Surgical	47	72	19	379	160	677	3,617
Total	220	107	188	449	161	1,125	5,990

**Table 2:** Screening for CLABSI

Patients with central line-associated bloodstream infections (CLABSIs)	
Screened for hospital acquired CLABSI	50
Excluded due to line placement prior to admission	32
Negative blood cultures	1
Positive blood culture prior to line placement	4
CLABSI hospital	13

**Table 3:** Demographics of patients meeting criteria for hospital-acquired CLABSI IJ - internal jugular; PICC - peripherally inserted central catheter; CLABSI - central line-associated bloodstream infection

Patient	Line type	# of line days	Temp 2 days before (+) blood cx	Temp 1 days before (+) blood cx	Tmax	WBC	Organism
1	Hemodialysis	19	99.8	99.1	99.6	16.3	Proteus mirabilis
2	Hemodialysis	92	98.4	99.8	102.9	27	Staphylococcus epidermidis
3	Femoral	36	97.6	97.5	97.8	13.6	Candida orthopsilosis
4	Subclavian	18	98.7	98.5	99.6	3.2	Enterococcus faecalis
5	Hemodialysis	28	98.8	98.7	100.6	20	Pseudomonas aeruginosa
6	PICC	8	98.9	99.8	101.2	0.2	Escherichia coli
7	Femoral	8	98.4	98.2	98.7	14.6	Enterococcus faecalis
8	Femoral	11	100.4	100.5	104	34.3	Staphylococcus aureus
9	IJ	27	98.9	99.8	101.2	7.3	Staphylococcus epidermis
10	Hemodialysis	5	97.5	99	99.7	3.9	Enterococcus faecalis
11	Right Femoral	40	99.9	100.1	102.5	16.3	Proteus mirabilis
12	PICC Line	47	98.9	99.8	101.2	0.2	Escherichia Coli
13	PICC Line	37	98.7	98.5	103.2	9.7	Staphylococcus aureus

**Table 4:** Central line infections by site and unit location PICC: peripherally inserted central catheter

Insertion site	ICU	Medical Surgical
Internal jugular	0/173 (0%)	1/47 (2.12%)
Subclavian	1/35 (2.86%)	0/72 (0%)
Femoral	2/169 (1.18%)	2/19 (10.53%)
PICC	0/70 (0%)	3/379 (0.79%)
Hemodialysis	1/1 (100%)	3/161 (1.88%)

**Table 5:** Line days in patients with and without CLABSI CVC - central venous catheter, CLABSI - central line-associated bloodstream infection

	# of CVC	Line days (mean)	Line days (total)
ICU			
CLABSI	4	23.25	93
No CLABSI	444	5.3	2,373
Medical Surgical			
CLABSI	9	31.66	284
No CLABSI	668	5.3	3,617

Patients with CLABSI had CVC inserted for a range of five to 92 days with the average number of line days being 29 days. The average temperature maximum for the patients with CLABSI was 101.1F with 64.3% of patients having a temperature greater than 100.4F. Normal number of white blood cells (WBC), defined as 4,500 to 11,000 WBCs per microliter, 92.3% of patients (12 of 13) had an abnormal WBC. Twelve of 13 patients were diagnosed with bacteremia (4/12 gram-positive, 8/12 gram-negative), while one was found to have Candida (Table 3).

CLIP compliance

Among all CVCs inserted, 58.6% (660/1125) had CLIP forms submitted. The rate of CLABSI from those CVC inserted with CLIP forms submitted was 61.5% (8/13).

## Discussion

In the present study, the rate of CLABSI in both the intensive care and medical-surgical units was 1.7 per 1,000 central line days and 2.8 per 1,000 central line days, respectively. This is triple the CLABSI rate in 2019, which was 0.69 per 1,000 line days. In 2020 during the COVID pandemic, the national average increased to 0.87 per 1,000 central line days in the ICU [1-3]. When accounting for all CVCs inserted at our institution (emergency department, operating room, interventional radiology, ICU, or floor units) rather than only those CVCs with submitted CLIP forms, the rate of CLABSI was significantly higher. The incidence CLABSI with CLIP form was 61.5%, and the incidence of CLABSI without CLIP form was 38.4%.

A higher prevalence of CLABSI was found with femoral and HD CVCs. When these were removed, the infection rate adjusted to 0.9 per 1,000 central line days - in line, on par with the national average. Femoral lines are well known to have higher rates of CLABSI, while SC lines are the least at risk for infection [5-8]. Furthermore, the CDC has recommended against the use of femoral central lines whenever possible in adult patients (Level of evidence: IA, BSI guidelines) and has recommended the use of the SC site in non-hemodialysis patients for short-term infusion (Level of evidence: IB) [9]. The preferred site for tunneled catheters has not been established, but the SC site should be avoided as hemodialysis patients with SC sites are at a higher risk of developing SC vein stenosis [9].

Concurrent CVC use is associated with nearly twofold risk of CLABSI compared with use of a single CVC [2]. Dube et al. reviewed 6877 patients with two or more days with concurrent or subsequent CVCs and found that 74 of 3,932 patients with concurrent CVC use (1.9%) developed CLABSI, compared with 81 of 7,864 patients with single CVC use (1.0%) [2]. Having two CVCs for longer than two-thirds of a patient’s CVC use duration was associated with increased likelihood of developing a CLABSI, adjusting for central line days and comorbidities. These lines were most frequently indicated for nutrition (14.1% of patients) or hemodialysis (43.4% of patients) [2].

The CLABSI rate was higher in patients in which CLIP forms were used (61.5%) compared to patients in which they were not used (38.4%). CLIP monitoring failed to capture 41.4% of CVCs inserted in our institution, 10.4% higher than estimated figures bythe National Healthcare Safety Network [10]. Non-compliance with CLIP form submission could be a source of preventable infection as it is associated with poor insertion practices and higher infection [10]. Although the rate of CLABSIs was higher in the CLIP form group, this is probably due to the fact that the infections were more appropriately documented.

Our institution’s poor CLIP form compliance was suggested to be due to the high volume of trauma cases and the emergent conditions under which CVCs are placed to resuscitate and stabilize trauma patients. Major trauma centers have been found to have higher rates of CLABSI [11]. In such cases, although adherence to best insertion practices may be difficult it must be required. In those patients with missing CLIP forms, we cannot assume that proper sterile technique was followed. Consequently, CVC without CLIP form compliance should be deemed a risk factor for CLABSI and these patients should have the CVC changed within 48 hours. When catheters are inserted under emergent conditions without CLIP forms, and the use of sterile technique cannot be ensured, the CDC recommends replacement of the catheter within 48 hours [9].

Proper documentation with checklists to ensure adherence to evidence-based guidelines has been shown to decrease CLABSI rates [12,13]. Failure to account for our institution’s non-compliance with CLIP forms may overestimate adherence to CLIP and its role in reducing CLABSI. Identifying low compliance with CLIP forms at our hospital highlights an area where significant improvements can be made to increase proper sterile insertion practices. This effort should be multidisciplinary, involving healthcare providers, infection control staff, nurses and unit managers. When stratified by line type, form submission, and adherence to CLIP elements, there is twofold higher compliance among PICC insertions [10]. Scott et al. found PICCs had more reliable line-day documentation. This was likely the effect of an optimized approach to insertion by a dedicated PICC team whereby CLIP form completion and insertion elements are included in the standardized protocol [10]. By contrast, all non-PICC lines were placed by resident or attending physicians who receive standardized insertion education, but are not necessarily required to ensure proper completion of this [10]. Quan et al. showed that CLIP form use and documentation regarding central line days improved from 55% to 99% when an electronic health record system was used to streamline workflow and CLIP elements were embedded into an electronic procedure note [14].

Based on the analysis of our CLABSI patient population, we suggest that the following changes could potentially help to decrease the rate of CLABSI: (1) that all patients receiving a CVC should adhere to CLIP documentation in all units, (2) that any femoral or HD CVC placed without a CLIP form should have the line changed within 48 hours, (3) that those patients with a femoral CVC or hemodialysis catheter in place for four days or greater with an abnormal WBC (<4.0 or >11 mg/dL) or abnormal temperature (<97.0F or >100.4F) should be considered for catheter exchange, and (4) that those patients with an IJ, SC, PICC CVC in place for seven days or greater with an abnormal WBC or abnormal temperature should have the catheter changed.

Future areas of study will include evaluation of line care practices given the average number of line days in patients with CLABSI. Another potential area to evaluate will be the duration that peripheral lines are in place at the time of a CLABSI.

Limitations

Our institutional review carried limitations. The biggest limitation was the inability to verify the total handwritten logged number of lines placed in the hospital with the electronic medical record system. Multiple attempts were made to collect the total number of CVC insertions performed at our institution using CPT codes and ICD-10 Procedure Codes, but this did not yield usable data. The total number of CVC insertions and line days were eventually determined using daily unit logs maintained by the unit managers. These unit logs proved to be highly accurate as they are maintained and updated daily by the unit managers. However, this limitation also makes it difficult to access in real-time CVC insertion and CLABSI to quickly improve line care practices.

## Conclusions

Our institutional review found a rate of CLABSI twice the national average in the ICU and triple the average in patients on the floor. We found poor compliance with CLIP form completion along with a higher prevalence in those with femoral and hemodialysis catheters. We propose a new set of criteria to reduce the number of CLABSI at our institution.

## Figures and Tables

**Figure 2:**
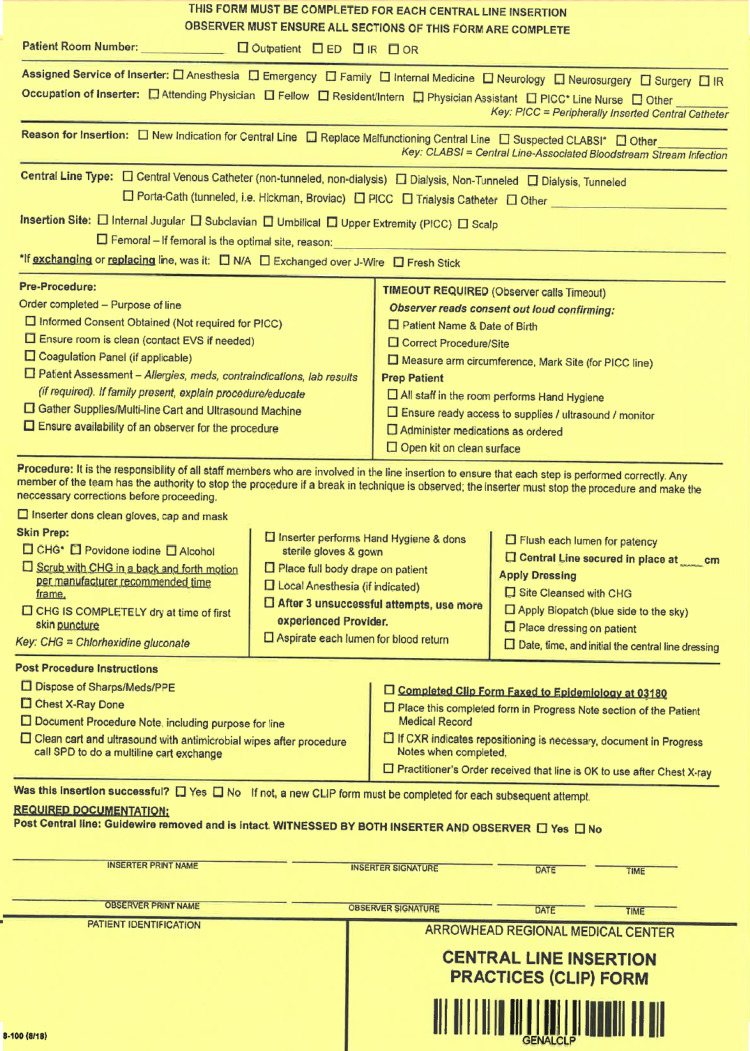
Institutional CLIP form
